# Wound of the Internal Iliac Artery, Caused by Instruments Used with Intent to Expedite Labor

**Published:** 1853-02

**Authors:** Wm. W. Morland

**Affiliations:** Sec’y.


From the Am. Journ. of Med. Sciences.

*Wound of the internal Iliac Artery, caused by Instruments used with intent to expedite Labor.*—(From "Extracts from the Records of the Boston Society for Medical Improvement. By Wm. W. Morland, M.D., Sec'y.")

Dr. Hayward, Sen., had seen, in a late number of the *London Times*, a report of a judicial action against a person accused of causing death by the manipulations above-mentioned.

In conjunction with this, Dr. H. referred to another case of the kind, which, singularly enough, he had heard an account of while in New Bedford, where it occurred, upon the same day on which he had read the report of the *Times*. A married lady, thirty-five years old, and six months advanced in pregnancy, went to the office of a homœopathic practitioner, who, it is supposed, at her request, attempted to procure abortion instrumentally; the membranes, however, as it subsequently appeared, were not ruptured; death followed; the operator is arrested and held for trial.

The following are the *post mortem* appearances, communicated to the Society by Dr. J. B. S. Jackson, who received them from Dr. Lyman Bartlett of New Bedford:—

*External Appearance of the Body.*—Surface very pale, otherwise normal; in left hypochondriac region an apparently recent contusion, near the superior spinous process of the ilium, in size about that of a nine-penny piece—yellow and hard; eleven other similar, but smaller spots below this, upon the abdomen and upper part of the left thigh; slight superficial ecchymosis, as from a contusion, about the posterior commissure of the labia externa; similar spots upon the nates, each side of the anus; evident ecchymotic contusion over the posterior portion of the labia externa; abdomen considerably distended.

*Dissection.*—Upon opening the *abdomen*, about two quarts of bloody serum escaped; the intestines were seen to be covered by an apron of coagulated blood, about six inches in vertical, and ten inches in transverse diameter, and of an average thickness of two inches; on removal of this coagulum, the intestines were seen, pale; the folds of the mesentery, attached to the lower portion of the small intestines, are separated by a clot of blood which would fill a pint bowl; the inferior fold of the mesentery was ruptured by the pressure of the blood accumulated between the folds. The *uterus* was flaccid, and in volume about the size of a three-pint bowl; on its posterior surface, opposite to the promontory of the sacrum, was observed an opening through its parietes, made, apparently, by some cutting instrument, whose diameter would be that of a pipe-stem, or of the common catheter; corresponding to this opening, another of the same size and appearance existed, going through the peritoneum forming the inferior fold of the mesentery. Beneath this puncture in the mesentery, and corresponding to it, was found an opening into the *right internal iliac artery*, one-fourth of an inch below the bifurcation of the main iliac artery, and of sufficient size to admit the point of a goosequill. On opening the uterus, a small fœtus was found, measuring seven and a half inches from the vertex capitis to the nates; its finger-nails perceptible. The membranes were not ruptured; on the posterior internal surface of the uterus, one and three-fourths inches above its cervix, was a puncture, extending obliquely two inches in length, through the parietes of the organ and terminating at the puncture previously mentioned, so that a blunt-pointed probe passed readily through from one orifice to the other. About half an inch from this puncture was found another, made obliquely into, but not going through, the uterine walls; its course parallel to the former; its length one and a half inches. Contusion, with ecchymosis, observed upon the internal surface of the posterior portion of the uterus.

All of these punctures corresponded with the mouth of the uterus, so that an instrument passed into the vagina would go in the direction, and produce the wounds, above described.

Death followed these manipulations in about twelve hours.

Present at the *post-mortem* examination, Drs. Spencer, A. and J. Mackie, Fulsome, and Lyman Bartlett.

